# QUALITY OF CONTACT WITH OLDER ADULTS AND KNOWLEDGE ABOUT AGING ARE ASSOCIATED WITH LOWER AGEISM AMONG YOUNG ADULTS

**DOI:** 10.1093/geroni/igz038.317

**Published:** 2019-11-08

**Authors:** Jessica H Helphrey, Cassidy M Adams, Leah N Smith, Jennifer Sawyer, Leigh A Fierro, Sarah Edzards, Allyson M Coldiron, Michael D Barnett

**Affiliations:** 1 The University of Texas at Tyler, Tyler, Texas, United States; 2 University of North Texas, Denton, Texas, United States; 3 The University of Texas at Tyler, Texas, United States

## Abstract

Ageism refers to stereotypes about and prejudice against individuals on the basis of age. Ageism among young adults may be different than other forms of intolerance simply because age changes; that is, young adults will grow older, and they will eventually become a member of what is presently an outgroup (i.e., older adults). The purpose of this study was to investigate whether ageism among young adults (N = 623) is more closely associated with future-oriented variables (i.e., optimism and fear of death) or whether ageism more closely resembles an outgroup attitude, which like other outgroup attitudes is mitigated by knowledge about and quality of contact with those outgroup members. Bivariate correlations found that knowledge of aging, quality of contact with older adults, and optimism were associated with lower ageism. In a multiple regression analysis, only knowledge about aging and quality of contact with older adults were associated with lower ageism. Overall, the results suggest that ageism represents more of an outgroup attitude rather than a future-oriented attitude. These results support the contact hypothesis in that knowledge of aging and quality of contact with older adults were associated with lower ageism among young adults. Education about aging and quality contact with older adults may be effective ways to reduce ageism among young adults.

